# cGAS: action in the nucleus

**DOI:** 10.3389/fimmu.2024.1380517

**Published:** 2024-03-07

**Authors:** Yikai Lu, Mengmeng Zhao, Li Chen, Yan Wang, Tianhao Liu, Haipeng Liu

**Affiliations:** ^1^ Central Laboratory, Shanghai Pulmonary Hospital, School of Medicine, Tongji University, Shanghai, China; ^2^ Research Center of Translational Medicine, Jinan Central Hospital Affiliated to Shandong First Medical University, Jinan, China

**Keywords:** cyclic GMP-AMP synthase (cGAS), nucleus, structure, activation, localization, functions

## Abstract

As a canonical cytoplasmic DNA sensor, cyclic GMP-AMP synthase (cGAS) plays a key role in innate immunity. In recent years, a growing number of studies have shown that cGAS can also be located in the nucleus and plays new functions such as regulating DNA damage repair, nuclear membrane repair, chromosome fusion, DNA replication, angiogenesis and other non-canonical functions. Meanwhile, the mechanisms underlying the nucleo-cytoplasmic transport and the regulation of cGAS activation have been revealed in recent years. Based on the current understanding of the structure, subcellular localization and canonical functions of cGAS, this review focuses on summarizing the mechanisms underlying nucleo-cytoplasmic transport, activity regulation and non-canonical functions of cGAS in the nucleus. We aim to provide insights into exploring the new functions of cGAS in the nucleus and advance its clinical translation.

## 1 Introduction

The innate immune system is the main contributor of the host defense against pathogen invasion and tissue damage (1). Pattern recognition receptors (PRRs) on innate immune cells recognize the pathogen-associated molecular patterns (PAMPs) carried by invading pathogens and the endogenous damage-associated molecular patterns (DAMPs) released by damaged cells, and then activates the innate immune response and prevents body damage (2, 3).

The discovery of cyclic GMP-AMP synthase (cGAS), the most interested PRRs in recent years, is a significant milestone in the field of DNA sensing, which plays a crucial role in infectious diseases (4), autoimmune diseases (5), cancers (6) and other diseases. cGAS recognizes both endogenous and exogenous DNA, such as pathogens DNA, mitochondrial DNA, micronuclei (MNi) DNA, genomic DNA, etc. (7, 8), then undergoes conformational changes and catalyzes the synthesis of the second messenger 2′3′-cyclic guanosine monophosphate–adenosine monophosphate (cGAMP) using adenosine triphosphate (ATP) and guanosine triphosphate (GTP) (9). cGAMP binds with stimulator of interferon genes (STING) and activates STING on endoplasmic reticulum (ER). Activated STING transfers from ER to Golgi apparatus (GA) and recruits TANK binding kinase 1 (TBK1) and IκB kinase (IKK) complex to activate interferon regulatory factor 3 (IRF3) and nuclear factor kappa-B (NF-κB), respectively. Subsequently, IRF3 and NF-κB enter the nucleus and induce the expression of cytokines, including type I interferon (IFN), which activate the innate immune response (10–13).

Since *Chen* team first reported cGAS in 2013, the biological function of cGAS in the cytoplasm has been widely studied (9). However, in recent years, a growing number of studies have shown that cGAS was also located in the nucleus (14–17). In 2018, our team first elucidated the nuclear function of cGAS in inhibiting DNA repair, which is dispensable of its canonical function in inducing STING-mediated type I IFN response (7). Subsequently, more STING-independent nuclear functions of cGAS were discovered, such as DNA damage repair (15), DNA replication regulation (16), chromosome fusion (17), etc. However, the mechanisms underlying nuclear localization, nucleo-cytoplasmic transport, activity regulation and nuclear functions of cGAS are still uncovered. Therefore, this review will briefly summarize the structure, activation, canonical function and subcellular localization of cGAS. Importantly, we focus on the mechanisms underlying nucleo-cytoplasmic transport, activity regulation and non-canonical functions of cGAS in the nucleus, in order to provide a more comprehensive understanding of nuclear cGAS. Meanwhile, we hope that this review could provide researchers with perspectives on the functions of nuclear cGAS and shed lights on clinical translational study by targeting nuclear cGAS.

## 2 Brief introduction of cGAS and its canonical function

### 2.1 Structural characteristics of cGAS

Human cGAS (hcGAS), also known as C6ORF150 or MB21D1, is a protein composed of 522 amino acids with a molecular weight of about 60 kDa, and belongs to the cGAS/DncV-like nucleotidyltransferase (CD-NTase) superfamily (8, 18). The structure of cGAS in *homo sapiens*, *sus scrofa* and other species is relatively conserved, containing a disordered N-terminal domain and a C-terminal catalytic domain (19, 20). The N-terminal domain, consisting of amino acids 1-160, contains a high density of positively charged amino acid residues that are necessary for binding double-stranded DNA (dsDNA). cGAS probably contains two different DNA binding sites, which respectively plays a role in the conformational changes of cGAS and the cooperative binding of cGAS dimers and DNA (21–23). The C-terminal domain, composed of amino acids 161-522, contains an NTase core structural domain, a highly conserved male abnormal 21 (Mab21) domain and a site-C dsDNA-binding domain (9, 24, 25). The NTase core domain and Mab21 domain constitute the two-leaf structure of the catalytic domain. The N-terminal lobe consists of two helices and a highly twisted β-folded NTase fold, which contains all catalytic residues (Glu225, Asp227, Asp319). The C-terminal lobe is a tightly helical bundle and contains a conserved zinc-ion binding module that mediates the binding with DNA and its dimerization. The margin of the deep groove between the two lobes constitutes the substrate binding site of the enzyme (19, 20). The site-C dsDNA-binding domain is mainly composed of three marker fragments: α-region (261–286), KRKR-loop (299-302) and KKH-loop (427-432). This domain promotes multivalency-induced liquid-phase condensation and cGAMP production. In addition, the positively charged residues of the three fragments mediate the interaction of cGAS with nucleosomal DNA, which contributes to the formation of the 2:2 cGAS-nucleosome complex (24, 25).

Liquid-liquid phase separation (LLPS) of intracellular biomacromolecules ensures the independence, high efficiency and precision of intracellular reactions (26, 27). Interestingly, the positive charged and disordered N-terminal domain of cGAS promotes the cGAS-DNA phase separation under the induction of negatively charged DNA. The cGAS-DNA phase separation creates a relatively independent environment and thus avoids being inhibited by negative regulatory factors, such as nucleic acid exonucleases three prime repair exonuclease 1 (TREX1) and barrier to autointegration factor (BAF) (7, 23). LLPS depends not only on intrinsic disordered region, but also on factors such as polyvalent molecular interactions, concentration, and environmental conditions (19, 23, 27). Because the valence of long DNA is higher than short DNA, long DNA is more effective in promoting the LLPS and the enzyme activity of cGAS (23). In addition, the concentration of cGAS and DNA in the cytoplasm also appears to be crucial for LLPS, and the immune response is initiated only when their concentration reaches a certain threshold (19, 28). A recent study reported that a DNA agglutination molecule called spermine promoted the condensation of naked DNA (including viral DNA) but not nucleosomal DNA to enhance and stabilize the binding of cGAS and DNA and then activate downstream immune response, thus providing a self-nonself recognition mechanism (29). Notably, LLPS of the cGAS-DNA complex is inhibited by viral tegument proteins such as ORF52 and VP22 of the γ- and α-herpesvirinae, by which viruses escape the immune surveillance (30).

### 2.2 Molecular mechanism of cGAS activation

As the canonical PRR, cGAS is activated by endogenous and exogenous DNA, including invaded pathogens DNA, mitochondrial DNA, MNi DNA, genomic DNA, etc (7, 8). At the structural level, cGAS typically preferentially binds to dsDNA and incomplete nucleoid-like or bent DNA. Due to the two substitutions (K187 and L195) in the DNA-binding surface, which are sufficient to direct preferential detection of long DNA, the affinity of cGAS and DNA depends on the length of DNA (>45 bp) rather than the DNA sequence specificity (31–34). Although single-stranded DNA (ssDNA) also binds with cGAS and induces limited cGAMP production, its binding affinity (Kd ~1.5 μM) is significantly weaker than dsDNA (Kd ~87.6 nM) (35). Interestingly, cGAS is also activated by unpaired DNA nucleotides flanking short base-paired DNA stretches in a structural and sequence-dependent manner. An example is the unpaired guanosines within the stem-loop structure of ssDNA derived from human immunodeficiency virus (HIV) type 1 (36). In addition, RNA : DNA hybrid strands also activate cGAS (37).

**Figure 1:**
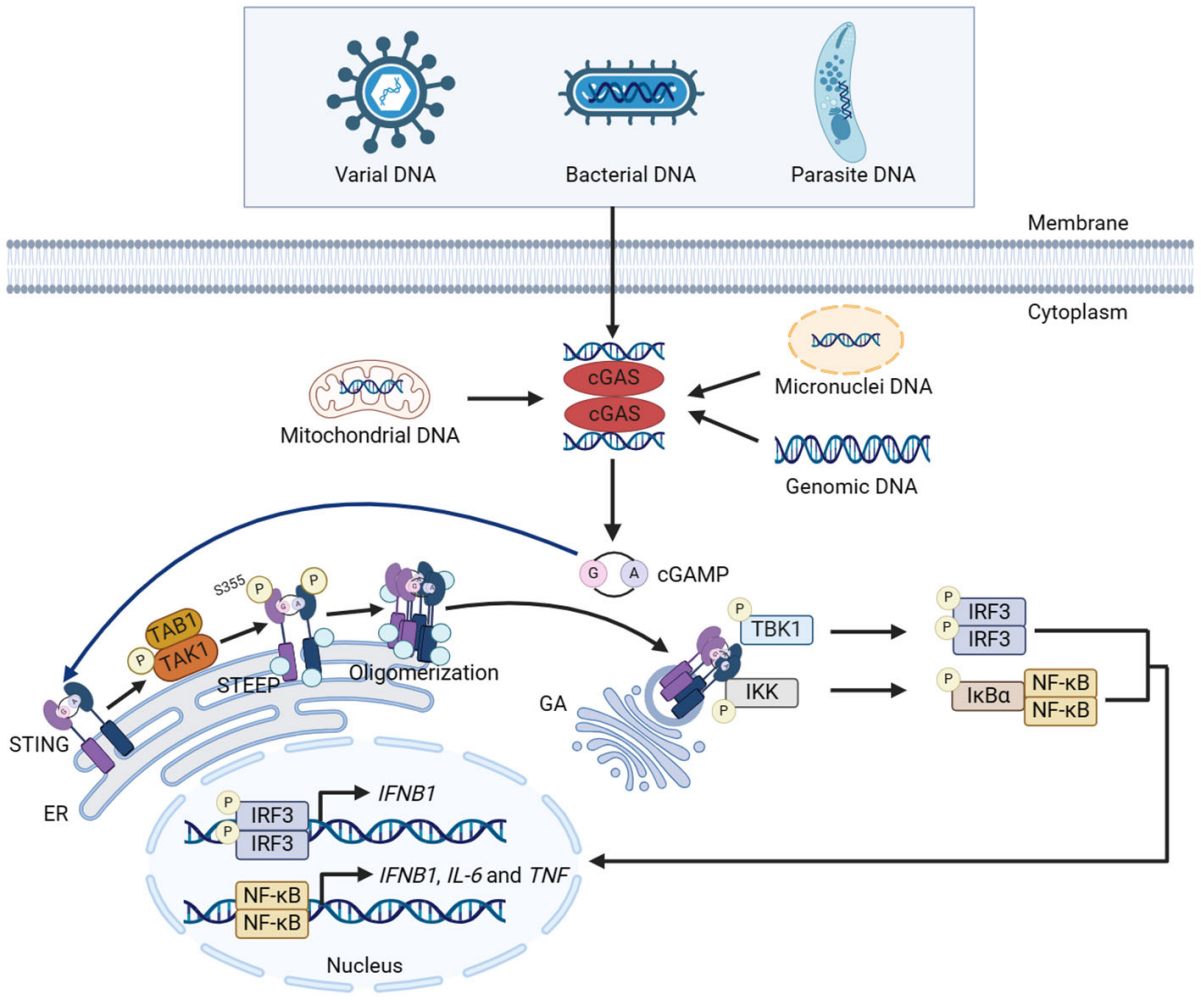
Canonical innate immune pathways induced by DNA. DNA from multiple sources induces the dimerization of cGAS and facilitates cGAS to catalyze the synthesis of cGAMP using ATP and GTP. cGAMP binds with STING and activates STING on the ER membrane. STING then activates TAK1, which directly mediates the phosphorylation of STING at S355 in a TAB1-dependent manner, facilitating the interaction between STING and STEEP. The interaction promotes STING oligomerization and translocation from ER to GA via ERGIC, recruiting and activating TBK1 and IKK complex. Activated TBK1 and IKK phosphorylate IRF3 and NF-κB, respectively, and promote the nuclear translocation of IRF3 and NF-κB, thereby inducing the expression of cytokines including type I IFN. The figures were created using scientific image and illustration software, BioRender (BioRender.com).

The conformational change of cGAS is induced by DNA binding. cGAS binds to the two phospho-sugar main chains of DNA, while the DNA double strand binds to the platform between the spine-like α helix and zinc finger structure of cGAS, forming a 2:2 dimer or highly ordered complexes. The cGAS dimers then form a ladder network structure, positioned head to head next to the DNA, resulting in the activation of cGAS (20, 21). After activated, cGAS performs the enzymatic function, catalyzing the synthesis of cGAMP using ATP and GTP (22). As a second messenger, cGAMP binds and activates STING on the ER membrane (38, 39). In the inactive state, STING is anchored to ER in the form of a ‘V’ dimer, and its C-terminal domain (CTD) is exposed in the cytoplasm. The CTD of the activated STING dimers rotates 180° and unwraps around the connector between proteins (including a connector loop and a connector helix), forming a more closed lid-like structure covering the cGAMP binding site (40, 41). STING then activates transforming growth factor beta-activated kinase 1 (TAK1) prior to STING trafficking in a TAK1 binding protein 1 (TAB1)-dependent manner. The activated TAK1 directly mediates the phosphorylation of STING at S355, facilitating its interaction with STING ER exit protein (STEEP), thus promoting STING oligomerization and translocation to ER-Golgi intermediate compartments (ERGIC) for subsequent activation (42). Subsequently, STING is transferred from ER to GA via ERGIC, recruiting and activating downstream factors such as TBK1 and IKK complex (43–45). Activated TBK1 phosphorylates the CTD of STING, and the phosphorylated CTD recruits IRF3 through its conserved and positively charged phospho-binding domain. Recruited IRF3 is phosphorylated by TBK1 and forms a dimer. The activated IRF3 translocates into the nucleus and functions as a transcription factor, thereby inducing the expression of type I IFN such as IFN-β (11, 45, 46). The activated IKK complex phosphorylates NF-κB inhibitor IκBα, which promotes the translocation of NF-κB into the nucleus and induces the expression of pro-inflammatory cytokines such as interleukin-6 (IL-6) and tumor necrosis factor (TNF) (11). IKK complex is composed of kinases IKKα and IKKβ, along with the regulatory subunit IKKγ (44, 47). IKKβ plays a key role in the activation of NF-κB mediated by cGAS-STING (44). Studies have shown that tripartite motif-containing protein (TRIM) 32 and TRIM56 is activated by cytoplasmic DNA, which add ubiquitin chains to NEMO and activate IKKβ, leading to the activation of NF-κB (44). Interestingly, the activated IKKβ is required for NF-κB and TBK1-IRF3 activation. Meanwhile, TBK1 is a reciprocal requirement for NF-κB activation, possibly by directly phosphorylating IKKβ. Thus, NF-κB/IRF3 activating cascade in which TBK1 and IKKβ forms a positive-feedback loop promotes the robust production of cytokines during cGAS-STING activation (44). Opposite to TRIM56 and TRIM32, TRIM29 has been reported to inhibit the innate immunity response. TRIM29 induces K48-linked ubiquitination of STING and promotes the degradation of STING, thus inhibiting the activation of innate immune response (**Figure 1**) (48).

### 2.3 Canonical functions of cGAS

Abnormal distributed nucleic acid resulting from pathogen invasion or unstable genome induced by DNA damage caused by various stimuli is recognized by cGAS, and then initiates the canonical innate immune response. The role of cGAS on the innate immune regulation was first found in pathogen infection and autoimmune diseases (9). dsDNA from a variety of pathogenic microorganisms such as *mycobacterium tuberculosis* (49), adenovirus (50) and *plasmodium falciparum* (51) is recognized by cGAS, which then promotes the expression of type I IFN and proinflammatory cytokines to enhance the anti-infection immunity. However, not all the canonical functions of cGAS are favorable for defensing infection. It has been reported that the host usually inhibits cGAS-induced type I IFN expression through promoting the production of caspase-1, which avoids the excessive production of type I IFN and decreases the host’s resistance to *mycobacterium bovis* infection (52). Studies have shown that cGAS/STING-deficient mice showed stronger resistance to *schistosoma mansoni* (53) and lethal *plasmodium yoelii* YM (54), while showed no difference on the scavenging of adenovirus (55). Interestingly, cGAS-deficient mice show higher levels of IFN-α and IFN-β *in vivo* when infected with lethal *plasmodium yoelii* YM (54).

Hyperactivity of cGAS-STING pathway may lead to sustained immune response and result in autoimmune diseases, such as systemic lupus erythematosus (56), Aicardi-Goutières syndrome (29), STING-associated vasculopathy with onset in infancy (57), rheumatoid arthritis (58) and psoriasis (59). To maintain the immune balance, the body also evolves various negative regulatory mechanism to avoid the hyperactivation of innate immunity. As a 3’-5’ exonuclease anchored to ER, TREX1 degrades MNi DNA and cytoplasmic DNA in time, thereby inhibiting the hyperactivation of cGAS (60, 61). In addition, autophagy-related gene 9a (62), Unc-51-like autophagy activating kinase 1 (63), NOD-like receptor family CARD domain containing 3 (64) and other proteins inhibit the activation of STING, thus avoiding excessive activation of the cGAS-STING pathway. Therefore, the negative regulation of the cGAS-STING pathway is of great significance for immune homeostasis.

The cGAS-STING pathway also plays a vital role in tumorigenesis and tumor development. cGAS-STING pathway not only inhibits tumor progression, but also promotes tumor progression. On the one hand, cGAS-STING pathway plays an anti-tumor role in melanoma (65), small cell lung cancer (66), breast cancer (67, 68) and colon cancer (69) by promoting the activation of immune cells, cell apoptosis and tumor vascular remodeling. On the other hand, cGAS-STING pathway promotes tumor progression in melanoma (70), breast cancer (71, 72), lung cancer (73) and skin cancer (74) by promoting tumor immune escape and metastasis and maintaining chronic inflammation. Surprisingly, proteins of oncogenic DNA viruses such as E7 of human papilloma virus and E1A of adenovirus, could bind with STING and inhibit the activation of cGAS-STING pathway. Specifically, these proteins promote tumor progression via antagonizing DNA sensing and blocking tumor suppressor factors. The specific proteins encoded by these viruses contain the LXCXE motif (50), which provides some implications for the antagonists of STING and the treatment of viral tumors.

## 3 Cellular localization of cGAS

The precise localization of cGAS in cells is vital for regulating its function. However, cellular localization of cGAS remains controversial. With the further study of cGAS, a growing number of studies have shown that cGAS was not only located in the cytoplasm (9), but also located in the cytomembrane (75), MNi (76), stress granules (SG) (77), mitochondria (78), lysosome (79) and the highly anticipated nucleus (15). Therefore, it is vital to confirm the localization of cGAS under physiological and pathological conditions, which helps the prevention and treatment of pathogenic microbial infection, autoimmune diseases, tumors and other diseases.

### 3.1 Cytoplasm

*Chen* team first detected cGAS in the cytoplasmic extract of human monocytic THP-1 cells by western blot. In mouse fibroblast cell line L929 stimulated with interferon-stimulated DNA, cGAS co-locates with DNA in the cytoplasm observed by laser-scanning confocal microscopy (9). cGAS interacts with B-lymphoid tyrosine kinase (BLK) in the cytoplasm under physiological conditions, resulting in the phosphorylation of cGAS at Y215, which maintains the cytoplasmic localization of cGAS (15). Cytoplasmic localization of cGAS is conducive to the rapid identification of its own DNA in cells undergoing DNA damage or facing invading pathogens, and also prevents the recognition of its own DNA in the nucleus and organelles by cGAS (80, 81).

### 3.2 Nucleus

Actually, when cGAS was first found locating in the cytoplasm, it was also observed locating in the nuclear or perinuclear region by laser-scanning confocal microscopy (9). In recent years, an increasing number of studies have confirmed that cGAS can be located in the nucleus (14–17). Unexpectedly, the vast majority of cGAS is located in the nucleus, regardless of whether cells are rapidly dividing or post-mitosis (82). Under physiological conditions, endogenous cGAS is tethered tightly in the nucleus by a salt-resistant interaction and this tight tethering is comparable to that of histones in its strength (82). In chromatin tethering, nuclear cGAS is not activated by self-DNA due to the blocking of DNA binding sites by acidic patch (AP) formed by histones H2A and H2B (83). Moreover, the activity of nuclear cGAS is regulated both in nucleosome and non-nucleosome dependent way. The activity regulation and the functions of cGAS locating in the nucleus under physiological and pathological conditions will be described in part 4 and part 5.

### 3.3 Cytomembrane

In 2019, *Kagan* team made a groundbreaking discovery that cGAS, which was previously believed to be a cytoplasmic protein in the resting state, was located on the cytomembrane in human and mouse macrophages. Specifically, cGAS is located on the cytomembrane through the combination of its positively charged N-terminal domain with negatively charged phosphoinositide phosphate, thus preventing the recognition of self-DNA (75). However, the localization of cGAS on the cytomembrane via its N-terminal domain is debatable. Another study showed that the N-terminal domain 1-212 of cGAS encodes a cytoplasmic retention activity in interphase (84). This suggests that the cellular localization of cGAS through its N-terminal is influenced by cell type, cell cycle and other factors.

### 3.4 MNi

cGAS is also located to the MNi in the cytoplasm (76). MNi is formed due to the error of chromosome separation in the process of cell division, and consists of chromatin surrounded by micronuclear envelope (mNE). MNi is usually in a non-immunogenic state when it has an intact mNE, compact chromatin with intact nucleosome and supercoiled DNA. Once these conditions are damaged, the immunogenicity of MNi is enhanced, resulting in recognition of MNi DNA by cGAS. Such immunogenic transformation is manifested in various forms, such as the rupture of mNE, the loss of nucleosome integrity, and the loosening of the supercoiled structures of MNi DNA (72, 76, 80). Recognition of MNi DNA by cGAS leads to the cGAS-STING cytoplasmic DNA sensing pathway and activates innate immunity (72, 76). In addition, cGAS serves as a micronucleophagy receptor for the clearance of MNi, maintaining MNi homeostasis to avoid hyperactivation of innate immunity within the cells. To be specific, cGAS directly interacts with microtubule associated protein 1 light chain 3 beta (MAP1LC3B/LC3B) through its MAP1LC3-interacting region located at amino acid sites 355-360, by which cGAS promotes the micronucleophagy and degradation in lysosome (85).

### 3.5 SG

cGAS is also located in SG (77). SG is formed by untranslated messenger ribonucleoproteins resulting from mRNA stalled in translation initiation, and is a subcellular structure regulating the RNA localization, translation, and degradation (86). Studies have shown that Ras–guanosine triphosphatase activating protein Src homology 3 domain–binding protein 1 (G3BP1), an important protein of SG, interacted with cGAS and engaged cGAS in a primary liquid-phase condensation state. Under the stimulation of exogenous DNA, the pre-condensed cGAS forms LLPS more efficiently, which promotes the rapid binding of cGAS and DNA and induces the expression of IFNs (77, 86, 87). Intriguingly, it is reported that RNA regulates cGAS activity by modulating the formation of cGAS-containing condensates (88). Therefore, it is worth considering whether PKR dependent RNA plays a role in the process of cGAS pre-condensation induced by G3BP1. In conclusion, according to the existing studies, SG is considered as a hub of nucleic acid receptors that promotes the recognition of exogenous nucleic acids and the initiation of innate immunity response.

### 3.6 Mitochondria

cGAS has been shown to recognize mitochondrial DNA (mtDNA) released by damaged mitochondria in the cytoplasm (78). Interestingly, damaged mtDNA could synergizes with MNi to activate cGAS and induce a robust immune response (80). However, the localization of cGAS on mitochondria, as well as its mechanism of localization on other subcellular organelles, remains unclear. Fortunately, a recent study has shown that cGAS is located on the outer mitochondrial membrane in hepatocellular carcinoma cells. Specifically, cGAS is translocated to mitochondria via the mitochondrial targeting sequence with the assistance of outer mitochondrial membrane 70, and interacts with dynamin-related protein 1 to promote oligomerization. This interaction inhibits the accumulation of mitochondrial ROS and ferroptosis in cancer cells, which in turn promotes cancer progression (78).

### 3.7 Lysosome

In 2019, *Chen* team discovered that the primordial function of the cGAS-STING pathway autophagy induction via STING trafficking independent of the TBK1 activation and IFNs production (89). In 2020, a study on the role of cGAS in promoting inflammation and autophagy in huntington disease (HD) mentioned the localization of cGAS in lysosomes in HD-homo striatal cells (79). Therefore, it is worth considering whether cGAS is regulated in lysosomes in a STING-dependent but TBK1-independent way.

## 4 Distribution of cGAS in the nucleus and regulation of its activation

In the past, researchers observed that cGAS could not be activated by genomic DNA, and the widely accepted explanation for this was that cGAS was mainly distributed in the cytoplasm, where it did not have access to genomic DNA. However, in recent years, several studies have reported that endogenous cGAS is widely present in the nucleus in resting states (14, 82, 90, 91). It has been found that endogenous cGAS was tightly tethered in the nucleus by a force with significant resistance to salt extraction. Therefore, the presence of nuclear cGAS was omitted from the conventional cytoplasmic and nuclear extracts (89). These findings make the study of the function and mechanism of nuclear located cGAS become a research hotspot in this field.

### 4.1 Distribution of cGAS in the nucleus

Endogenous cGAS is tethered tightly in the nucleus and sometimes located to ‘chromatin bridges’ between adjacent cells (82). The formation of ‘chromatin bridges’ is thought to be associated with the fusions of chromosome and the incomplete segregation of DNA between daughter cells during mitosis (82, 92). It has been shown that ‘chromatin bridges’ occurred when telomere fusions were induced in various cell lines, and nuclear envelope rupture during interphase occurred more frequently in cells with ‘chromatin bridges’. It is worth noting that telomere crisis is closely related to cancer (93). So what role does ‘chromatin bridges’ play in cancer? Is the localization of cGAS in the ‘chromatin bridges’ related to the nucleo-cytoplasmic localization, and is it closely related to the occurrence and development of cancer? These are urgent issues remaining to be addressed.

### 4.2 Nucleo-cytoplasmic transport of cGAS

**Figure 2:**
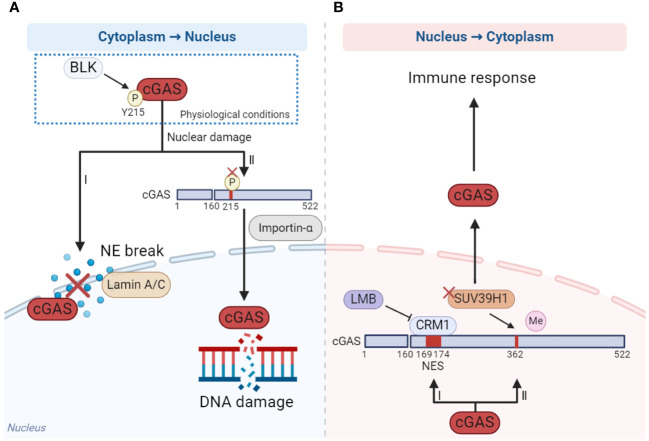
The mechanism of cGAS nucleo-cytoplasmic transport. **(A)** When DNA damage occurs, cGAS is dephosphorylated at Y215 and translocated from cytoplasm to the nucleus depending on importin-α (I), while the nuclear membrane is ruptured, the translocation of cGAS from cytoplasm to the nucleus is dependent on laminin A/C (II). **(B)** When cells are stimulated by cytoplasmic DNA, cGAS translocates from nucleus to cytoplasm in a CRM1-dependent manner under the effect of NES (I). In addition, reducing the methylation of cGAS by methyltransferase SUV39H1 promotes cGAS translocating to the cytoplasm (II). The figures were created using scientific image and illustration software, BioRender (BioRender.com).

Nucleo-cytoplasmic transport of cGAS is precisely regulated which ensures that cGAS performs its function in the proper localization. cGAS is mainly located in the cytoplasm in nondividing cells and in the nucleus in mitotic proliferating cells (90). According to the existing reports, cytoplasmic retention and accumulation of cGAS may depend on the phosphorylation of Y215 and N-terminal domains mentioned above (**Figure 2A**) (15, 84). However, cGAS is translocated from the cytoplasm to the nucleus once nuclear damage occurs. When cells undergo DNA damage, the Y215 of cGAS is dephosphorylated, leading to its translocation from the cytoplasm to the nucleus through importin-α-dependent mechanisms. Nuclear cGAS is then recruited to double-strand breaks (DSBs) points in damaged DNA, where it inhibits the formation of the PARP1-Timeless complex through poly(ADP-ribose) interaction with PARP1 and impairs homologous-recombination-mediated DNA damage repair (**Figure 2A**) (15). Another study has shown that cGAS relied on lamin A/C to translocate into the nucleus and gather at the rupture site of the nuclear envelope (NE) when the NE broke (**Figure 2A**) (94).

How does the nuclear cGAS translocate to the cytoplasm? When stimulated by cytoplasmic DNA, cGAS translocates from the nucleus to the cytoplasm in a chromosome region maintenance 1 (CRM1)-dependent manner, facilitated by the nuclear export signal (NES) locating at amino acid sites 169-174 of cGAS. This translocation activates immune response. Once the NES is mutated or the CRM1 is inhibited by inhibitor leptomycin B (LMB), cGAS could not enter the cytoplasm from the nucleus and the activation of innate immune including IFN response is significantly inhibited (**Figure 2B**) (95). In addition, reducing the methylation of cGAS by methyltransferase SUV39H1 can promote cGAS translocation into the cytoplasm and thus promote the activation of the immune response (**Figure 2B**) (96).

### 4.3 Inactivation mechanism of nuclear cGAS

In recent years, some explanations have been proposed to elucidate why cGAS is not activated by self-DNA in the nucleus. Currently, the inhibition of cGAS activity in the nucleus is mainly explained from two perspectives: non-nucleosome regulation (97) and nucleosome regulation (14).

#### 4.3.1 Non-nucleosome regulation

**Figure 3:**
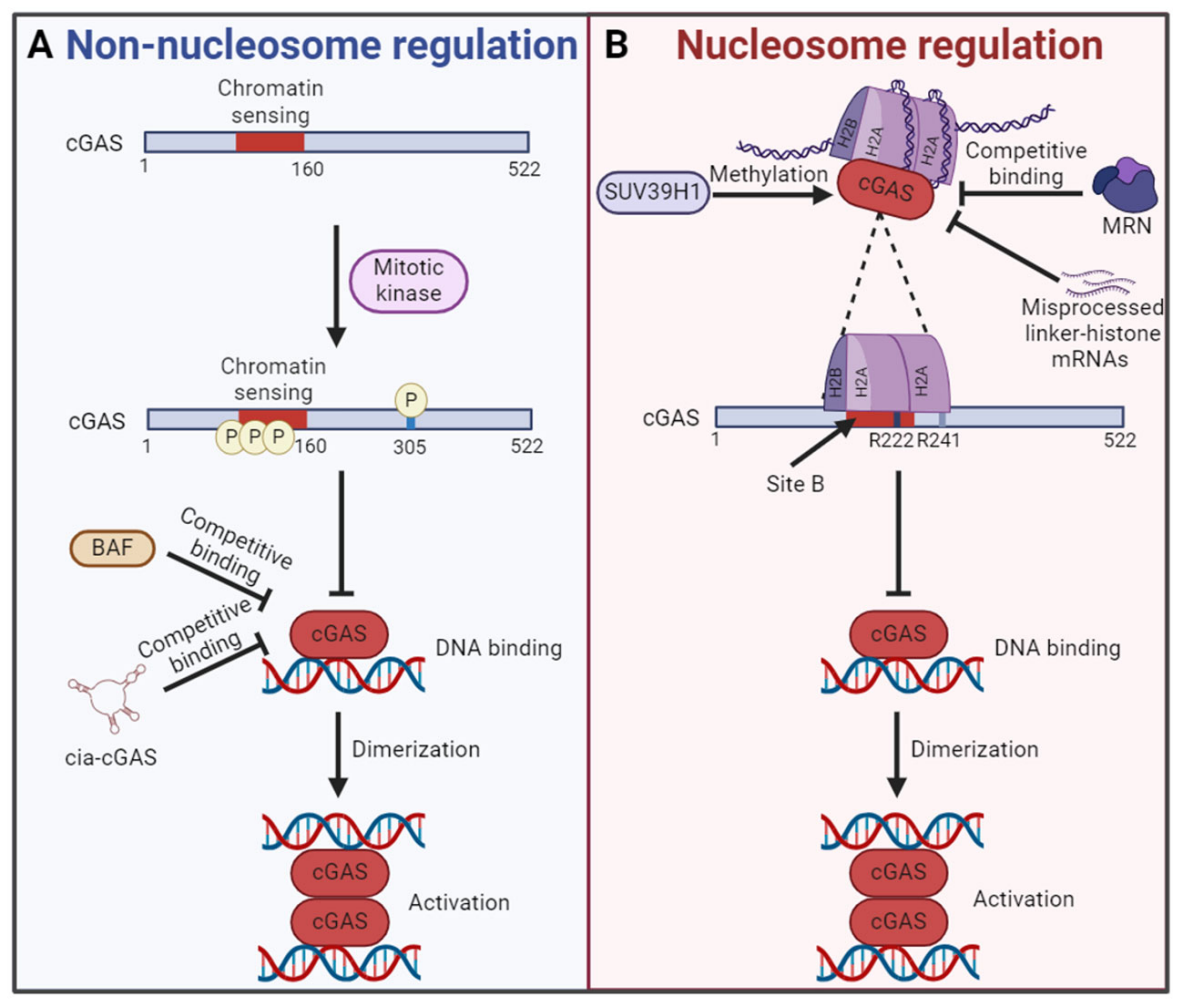
The mechanism of cGAS inactivation in the nucleus. **(A)** In nucleosome independent way, the N-terminal chromatin sensing region of cGAS or S305 is hyperphosphorylated by mitotic kinase. What’s more, the competitive binding of BAF or cia-cGAS to DNA also inhibits the cGAS activation. Both mechanisms lead to the failure of cGAS binding with self-DNA and the formation of cGAS dimers for activation. **(B)** In nucleosome dependent way, the DNA binding site B of cGAS is blocked by interacting with the AP. The AP is formed by histones H2A and H2B. Interact with the AP is cGAS conserved amino acid residues R222 and R241. In this regard, the chromatin tethering could be altered by the methylation of cGAS by SUV39H1, competitive binding of MRN and accumulation of misprocessed linker-histone mRNAs, etc. The figures were created using scientific image and illustration software, BioRender (BioRender.com).

In 2017, *Chen* team found that cGAS entered the nucleus during mitosis in proliferating cells and was associated with chromatin DNA, suggesting that cGAS may regulate cell cycle and cellular senescence through an unrevealed mechanism (90). Further studies show that cGAS activity is selectively inhibited during mitosis in human cell lines and reveal two parallel mechanisms for this inhibition, both of which are dispensable of nucleosome. On the one hand, the N-terminal domain of cGAS is hyperphosphorylated by mitotic kinases, including Aurora kinase B, which leads to the blocking of chromatin sensing. On the other hand, oligomerization of chromatin-bound cGAS is prevented, leading to the inhibition of cGAS activation. These two mechanisms ensure that cGAS is inactive upon entry into the nucleus during mitosis, which may help prevent autoimmune responses (**Figure 3A**) (97). Also during mitosis, human cGAS is phosphorylated by mitotic cyclin-dependent kinase 1 (CDK1)-cyclin B complex at the highly conserved S305, and phosphorylation at this site inhibits cGAS activity and cGAMP synthesis (**Figure 3A**). However, the self-DNA sensing capability of cGAS is restored when cGAS is dephosphorylated by type 1 phosphatase PP1 at mitotic exit (98). In addition, BAF competitively binds self-DNA with cGAS during acute loss of nuclear membrane integrity, thereby limiting cGAS activation (**Figure 3A**) (99). Moreover, circular RNA antagonists cia-cGAS binds nuclear cGAS to avoid the detection of self-DNA and blocks its enzymatic activity, thus maintaining homeostasis of hematopoietic stem cells (**Figure 3A**) (100). Interestingly, spermine, one of the three polyamines synthesized in mammals, plays an important role in cGAS distinguishing between self and non-self DNA. Spermine effectively promotes the condensation of viral DNA rather than host nucleosome DNA, which is important for the immune system to protect against pathogens and maintain tolerance to the host and innocuous commensals during homeostasis (29).

#### 4.3.2 Nucleosome regulation

Based on the discovery that cGAS was located in the nucleus and bound to chromatin (89), *Li* team found that the catalytic domain of cGAS promoted the binding of cGAS and nucleosome (14). The activity of cGAS in the nucleosome/cGAS complex is inhibited, which could not catalyze the synthesis of cGAMP. Interestingly, although oligonucleosome inhibits the activity of cGAS, a certain amount of cGAMP is synthesized when excessive cGAS is added to oligonucleosome/cGAS complex. The researchers suspect that the bare DNA at the junctions in the oligonucleosome may be responsible. The naked DNA activates cGAS that is not bound to the nucleosome (14). Further studies reveal that cGAS does not interact with nucleosome DNA. Actually, cGAS interacts with a negatively charged AP formed by histones H2A and H2B via its conserved amino acid residues R222 and R241 with nanomolar affinity. This interaction blocks the second DNA binding domain (site B) of cGAS and inhibits the formation of cGAS dimers, resulting in cGAS maintaining in an inactive conformation (14, 92). These findings confirm the critical role of nucleosome in inhibiting the activation of cGAS in the nucleus, which is a major breakthrough for the study of self and non-self discrimination of genomic DNA by cGAS (**Figure 3B**). Interestingly, it has been found that competitive uptake of methionine by tumor cells activated the methyltransferase SUV39H1 and promoted the methylation of cGAS, thus promoting the chromatin tethering of cGAS without activation (**Figure 3B**) (96). However, the chromatin tethering could be altered by molecular displacement. A recent study demonstrated that binding of the MRE11-RAD50-NBN (MRN) complex to nucleosome fragments was essential to displace cGAS from AP-mediated sequestration, which enabled its mobilization and activation by dsDNA (**Figure 3B**) (101). In addition, the chromatin tethering could also be disrupted by the accumulation of misprocessed linker-histone mRNAs and histone stoichiometry alteration, which are caused by mutations in the LSM11 (U7 snRNA-associated Sm-like protein) and RNU7-1 (RNA, U7 small nuclear 1) genes encoding core components of histone mRNA-preprocessing complex (**Figure 3B**) (102).

## 5 Functions of nuclear cGAS

### 5.1 DNA damage repair

**Figure 4:**
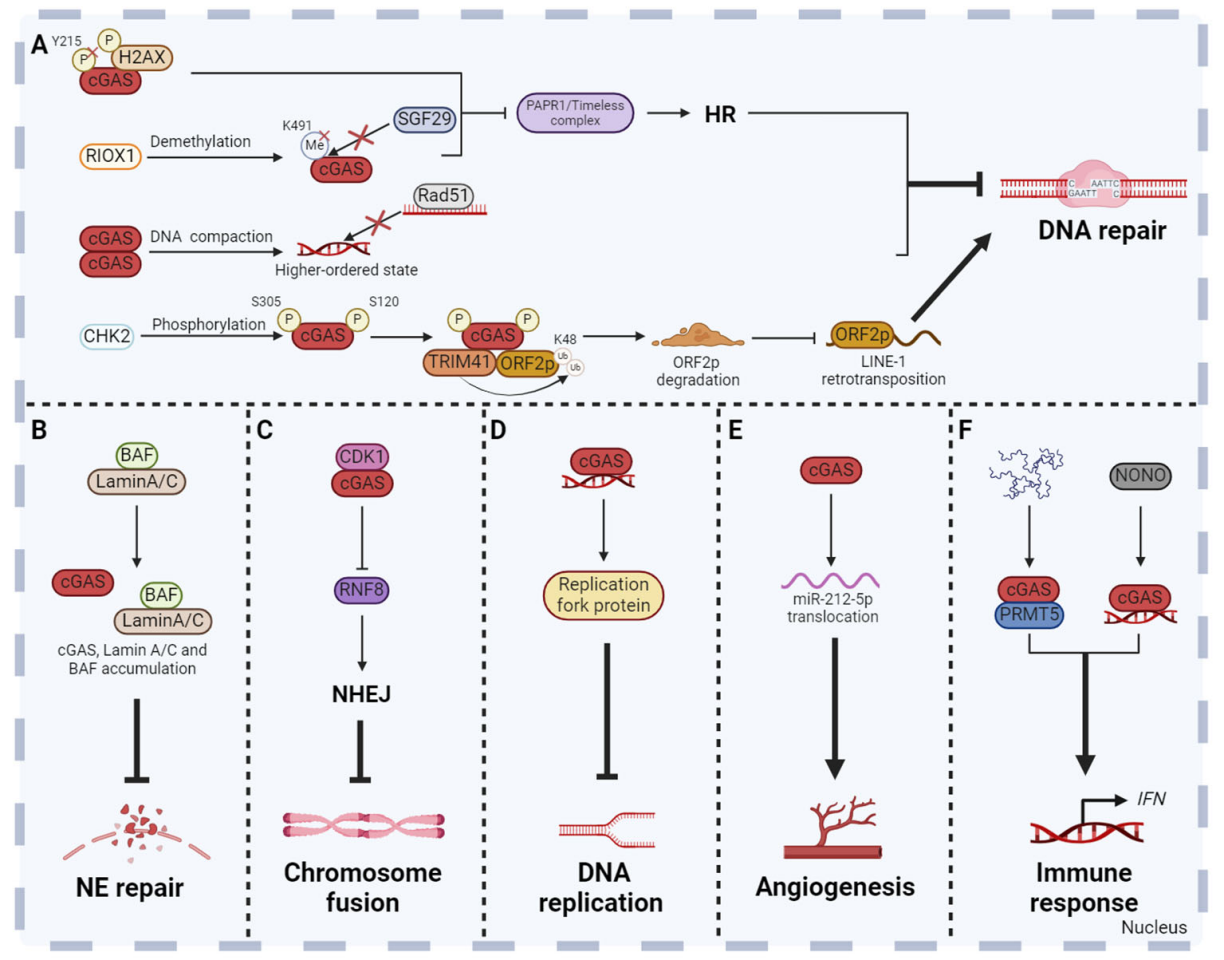
Non-canonical functions of cGAS in the nucleus. Besides the DNA sensor function, cGAS has been revealed many new functions in the nucleus. Nuclear cGAS plays a role in DNA repair **(A)**, NE repair **(B)**, chromosome fusion **(C)**, DNA replication **(D)**, angiogenesis **(E)** and non-cytoplasmic innate immune response **(F)**. These functions collectively enhance the host defense, inflammatory response and anti-tumor immunity, so as to maintain human physiological health. The figures were created using scientific image and illustration software, BioRender (BioRender.com).

There are many DNA damage factors that affect human health both *in vivo* and *in vitro*. For example, DSBs are potentially highly harmful. If not properly repaired, DSBs can lead to chromosomal deletion or translocation, which eventually result in diseases related to genomic instability, including tumorigenesis, accelerated aging, and other diseases (103). Powerful DNA damage repair mechanisms ensure the integrity of DNA. According to current reports, there are at least five major DNA damage repair pathways, including non-homologous end joining (NHEJ), base excision repair (BER), nucleotide excision repair (NER), mismatch repair (MMR) and homologous recombination (HR) (104). Currently, most studies have proved that cGAS inhibited homologous recombination in response to DNA damage induced by different conditions and thus inhibited DNA damage repair (15, 105–107). Our study demonstrated that nuclear cGAS contributes to tumorigenesis by inhibiting HR-mediated DNA damage repair. Once the DNA damage occurred, cytoplasmic cGAS was dephosphorylated at Y215 and translocated into the nucleus. The C-terminal domain of nuclear cGAS is recruited to DNA damage sites after binding to phosphorylated H2AX and inhibits the formation of PAPR1/Timeless complex, thereby inhibiting HR and increasing genomic instability (15). Under ionizing radiation, demethylase ribosomal oxygenase 1 (RIOX1) removes monomethylation at K491 of cGAS, which promotes the separation of cGAS and methyl-lysine reader protein SAGA complex-associated factor 29 (SGF29). The released cGAS binds to PAPR1 to inhibit the formation of PAPR1/Timeless complex, thereby inhibiting HR (105). Moreover, nuclear translocated cGAS inhibits HR-mediated DNA damage repair and promotes mutagenesis in human hepatocytes after microcystiin-LR (MC-LR) treatment, while BLK inhibits nuclear translocation of cGAS and cell mutagenesis induced by MC-LR (106). Besides the important role of the cGAS-PARP1-Timeless axis in HR suppression, cGAS also inhibits HR through other mechanisms. Another study found that nuclear cGAS dimerization impaired HR efficiency by compacting dsDNA into a higher‐ordered state. This state hindered RAD51-coated ssDNA filaments invasion, thereby affecting D-loop formation during HR-mediated DNA damage repair. Eventually, it leads to genomic instability, the generation of MNi and cell death under genomic stress conditions (**Figure 4A**) (107). In addition, cGAS has also been reported to prevent excessive DNA damage by inhibiting long interspersed nuclear element (LINE, also called long retroposon)-1 (LINE-1) retrotransposition, thereby maintaining genomic stability (**Figure 4A**) (81, 108). It has been reported that cGAS was enriched in the LINE region in the nucleus (81, 109), and recognized the complementary DNA produced by reverse transcription of LINE-1 to induce pro-inflammatory cytokines production (110). However, whether nuclear cGAS directly participates in the regulation of LINE retrotransposition and the underlying mechanism have not been characterized. ORF2p, a protein encoded by the open reading frame in full-length LINE-1, is crucial for LINE-1 retrotransposition and causes DNA strand breaks which poses a threat to genome integrity via its endonuclease activity (111–113). Recently, *Mao* team revealed that upon the occurrence of DNA damage, the checkpoint kinase CHK2 interacted with and phosphorylated nuclear cGAS at S120 and S305 residues to facilitate the interaction of cGAS-TRIM41 and TRIM41-ORF2p, which promoted TRIM41-mediated ORF2p ubiquitination and degradation and restricted LINE-1 retrotransposition, thereby maintaining genomic stability (108).

### 5.2 NE repair

Due to the weakening of the NE structural integrity, leakage of nuclear proteins containing a nuclear localization signal (NLS) into the cytoplasm has been observed under normal physiological and pathological conditions. However, the NE repair process is essential to prevent nuclear dysfunction due to accumulation of DNA damage and leakage of macromolecules into the cytoplasm (94, 114, 115). When NE is ruptured, laminin C rapidly accumulates at the site of rupture, which requires both the immunoglobulin-like fold domain that binds to BAF and the NLS. In addition, cGAS also co-aggregates with laminin C and BAF at this site, partly dependent on laminin A/C to facilitate rapid repair (**Figure 4B**) (94).

### 5.3 Chromosome fusion

Cellular senescence is a self-defense mechanism activated by intrinsic stimuli and/or exogenous stress, and is one of the central hallmarks of senescence (116). There are many factors inducing cellular senescence, including telomere attrition, loss of tumor suppressors, mitochondrial dysfunction, perturbed proteostasis, autophagy impairment, cytokines, oxidative stress, nutrient deprivation, epigenetic modifiers, genotoxic drugs and other internal and external factors. Senescent cells secrete a large number of factors, including IL, chemokines, growth factors, non-protein molecules, insoluble factors and so on, collectively termed the senescence messaging secretome (SMS) or senescent associated secretory phenotype (SASP) (117). It has been shown that the cellular senescence is inhibited in cGAS-deficient cell which accelerates spontaneous immortalization. Meanwhile, the SASP induced by DNA damage is also suppressed (90). cGAS activation and STING dimer formation were found in senescent cells, suggesting that the cGAS-STING pathway is involved in the regulation of cellular senescence (118). In addition, cGAS also regulates cellular senescence in a STING-independent manner through inhibiting chromosome fusion. Chromosome fusion is a manifestation of chromosomal instability, usually caused by the loss of telomeric repeat sequences or deficiencies in telomeric proteins (119). Recently, further studies revealed that cGAS could interact with CDK1 and localize them to chromosome ends (17). CDK1 then blocks RNF8 recruitment to inhibit NHEJ-mediated DSBs repair during mitosis. This process contributes to reducing the occurrence of end fusions on broken chromosomes and facilitating replicative senescence (17). However, the absence of cGAS can lead to problems in cellular senescence, showing chromosome end-to-end fusions, genomic instability, and long-term growth arrest (17). Cancer cells often have a high rate of spontaneous telomere loss, resulting in chromosome fusion and other chromosomal instability (119). The mechanism of cGAS inhibiting chromosome fusion and promoting replicative senescence may be a vital breakthrough in tumor therapy (**Figure 4C**).

### 5.4 DNA replication

Complete and accurate DNA replication is an important basis for cell proliferation and genome stability. Any errors of DNA replication will result in abnormal DNA replication, which can lead to diseases such as tumors. Compared to normal cells, the replication forks, replication origin, S-checkpoint and other replication stress are abnormal in tumor cells, which results in genomic instability (120). *Lan* team found that cGAS-deficient untransformed cancer cells exhibited uncontrolled DNA replication, leading to genomic instability and excessive proliferation (16). Further mechanism research found that cGAS promoted the genomic stability by acting as a ‘decelerator’ for DNA replication (16). cGAS interacts with replication fork proteins in a DNA binding-dependent manner in the nucleus to slow replication fork movement and reduce cell susceptibility to DNA damage, indicating that cGAS is an attractive target for exploiting genomic instability of cancer cells (16). It is deserved to be mentioned that STING is often inhibited in numerous cancers, which contributes to the resistance to tumor immunity (121). The new STING independent function of cGAS in replication dynamics is expected to be a potential therapeutic target for STING deficient tumors (**Figure 4D**).

### 5.5 Angiogenesis

Angiogenesis is the formation of new blood vessels from the existing vascular system, which is a dynamically regulated biological event (122). Excessive or insufficient angiogenesis has been linked to diseases such as cancer and diabetes (123). Interestingly, nuclear cGAS regulates vascular endothelial growth factor-A (VEGF-A)-mediated angiogenesis in an immune-independent manner (124). Mechanism studies showed that VEGF-A stimulation induced cGAS nuclear translocation through importin-β pathway. Subsequently, nuclear cGAS regulates the miR-212-5p-ARPC3 cascade to influence cytoskeletal dynamics and VEGF receptor 2 trafficking from the trans-Golgi network to the cytomembrane (124). This study suggests that cGAS may be a potential therapeutic target for pathologic angiogenesis related diseases (**Figure 4E**).

### 5.6 Non-cytoplasmic innate immune response

In fact, cGAS plays the role in innate immune response not only in the cytoplasm, but also in the nucleus. Nuclear cGAS enriched in centromeric satellite DNA and LINE synthesizes cGAMP and induces innate immune response in primary human monocyte derived dendritic cells (DCs). The level of cGAMP synthesized by nuclear cGAS is 200 times lower than that stimulated with exogenous DNA (84). This non-canonical innate immune response is usually caused by various nuclear pathogens, but the mechanism of cGAS activation is different. HIV activates nuclear cGAS through the interaction of nuclear cGAS and non-POU domain-containing octamer-binding protein in DCs and macrophages (125). Herpes simplex virus 1 infection results in the release of cGAS from chromatin into the nuclear soluble fraction. Released nuclear soluble cGAS senses viral DNA and produces cGAMP, which subsequently induces the expression of type I IFN and IFN-stimulated genes (126). Moreover, nuclear cGAS interacts with protein arginine methyltransferase 5 (PRMT5) to catalyze the symmetric dimethylation of the histone H3 arginine 2 at *Ifnb* and *Ifna4* promoters, thereby promoting the entry of IRF3 and inducing IFN production (**Figure 4F**) (127).

## 6 Conclusion

Currently, our understanding of the role of cGAS as a DNA sensor has significantly advanced. Cytoplasmic cGAS is a crucial receptor for both endogenous and exogenous DNA which induces the activation of innate immune response and serves as a master regulator in inflammation, senescence, cancer and so on, making it an appealing target for the drug development (10, 128). Moreover, several important questions that have been puzzling researchers in this field have been more or less answered. Why is cGAS not activated by the DNA in the nucleus? What function it plays in the nucleus (14, 15, 97)? However, there are still a variety of interesting questions to be addressed. First, the localization of cGAS has long been controversial. cGAS is not only located in the cytoplasm as previously reported, but also located in the nucleus and cytomembrane (15, 75). It remains challenging to clarify the mechanisms underlying the tight regulation of the localization of cGAS. Second, cell cycle has been implicated to be correlated with the activity of cGAS (97), the exact underlying mechanism warrants further clarification. Third, it’s now well accepted that the enzymatic activity of cGAS can be inhibited by binding to chromatin nucleosome in the nucleus (14), however, whether cGAS participates in transcriptional regulation through modulating chromatin accessibility is unknown. LLPS has been previously reported to be important for regulating the activity of transcriptional factors such as IRF1 (129), and cGAS is capable of forming LLPS (23). Therefore, it’s interesting to question whether LLPS of cGAS serves as a mechanism underlying transcriptional regulation. It remains attractive to further characterize the novel function of cGAS in the nucleus. In a word, the study of the function of cGAS in the nucleus is still urgently needed, which may thereby provide the basis for drug development to precisely target nuclear cGAS.

## Author contributions

YL: Writing – original draft, Writing – review & editing. MZ: Writing – original draft, Writing – review & editing. LC: Writing – review & editing. YW: Writing – original draft. TL: Writing – original draft. HL: Writing – original draft, Writing – review & editing.
